# Determinants of rural-urban differential in healthcare utilization among the elderly population in India

**DOI:** 10.1186/s12889-021-10773-1

**Published:** 2021-05-17

**Authors:** Shreya Banerjee

**Affiliations:** Centre for the Study of Regional Development, School of Social Sciences, Jawaharlal Nehru University, New Delhi, India

**Keywords:** Rural-urban disparity, Elderly population, Health inequality, Decomposition analysis, Demographic burden

## Abstract

**Background:**

Population aging poses a demographic burden on a country such as India with inadequate social security systems and very low public investment in health sector. This challenge of accelerated demographic transition is coupled by the rural-urban disparity in access to healthcare services among the elderly people in India. An important objective of India’s National Health Policy (2017) is to *“progressively achieve universal health coverage”* which is posited upon mitigating the sub-national disparity that necessitates identifying the drivers of the disparity for targeted policy intervention. This study, therefore, makes an attempt towards the exploration of the prominent contributory factors behind the rural-urban gap in utilisation of healthcare among the older population in India.

**Methods:**

The analysis has been done by using the unit level data of Social Consumption: Health (Schedule number 25.0) of the 75th round of the National sample Survey conducted during July 2017–June 2018. Two binary logistic models have been proposed to capture the crude and the adjusted association between health seeking behaviour and place of residence (rural/ urban). To compute the group differences (between rural and urban) in the rate of healthcare utilization among the elderly population in India and to decompose these differences into the major contributing factors, Fairlie’s decomposition method has been employed.

**Results:**

The logistic regression models established a strong association between place of residence and likelihood of healthcare utilisation among the Indian elderly people. The results of the Fairlie’s decomposition analysis revealed considerable rural-urban inequality disfavouring the rural residents and health care utilisation was found to be 7 percentage points higher among the older population residing in urban India than their rural counterparts. Level of education and economic status, both of which are indicators of a person’s Socio-Economic Status, were the two major determinants of the existing rural-urban differential in healthcare utilisation, together explaining 41% of the existing rural-urban differential.

**Conclusion:**

Public health care provisions need to be strengthened both in terms of quality and outreach by way of greater public investments in the health sector and by building advanced health infrastructure in the rural areas. Implementation of poverty alleviation programmes and ensuring social-security of the elderly are also indispensable in bringing about equity in healthcare utilisation.

## Background

The phenomenon of population aging in India has emerged as a major concern for policymakers, researchers and other stakeholders. In light of the profound impact that the aging of population in India can potentially have on the social, political and economic spheres alike, research on the health and well-being of the elderly population is the need of the hour to ensure and facilitate the process of healthy aging. India has been experiencing a gradual increase in both the size and share of the older population over the past few decades. The elderly population has increased from 24.71 million in 1961 to 104 million in 2011, constituting 5.6% in 1961 to 8.6% of the total population in 2011 [1]. The older population in India is growing at a rate three times higher than the rate of growth of population as a whole [2]. This changing demographic landscape of India is largely attributed to an improvement in the longevity, among other factors like falling fertility [3]. Life expectancy at birth in India has increased from 42 years in 1961 to 69 years in 2018 and is projected to increase to 76 years by 2050 [4]. Likewise, both life expectancy at age 60 and 80 has improved considerably and stands at 18 and 7 years respectively, projected to rise to 21 and 8.5 years by 2050 [4]. However, the more important question that lingers is whether this increase in longevity has been accompanied by a commensurate improvement in health status and health care utilisation among the elderly population in India. On the one hand, while the increase in absolute numbers and share of older population in the country due to improved life expectancy is indicative of social, economic and epidemiological/ health achievements, on the other hand, a challenge of this demographic transition is manifested in the form of increasing old-age dependency ratio, that has increased from 5% in 1960 to 9% in 2018, projected to further rise steadily to 19% in the next three decades [4]. Clearly, population aging in India poses a demographic burden on a country such as India with inadequate social security systems and very low public investment in health and other welfare programmes [5].

Furthermore, the distribution of aging population in India, by place of residence, revealed that a vast majority (67%) of India’s older population resides in the rural areas [6]. At the country level, 27.72% of the older population reported an illness whether chronic^1^ or short-term^2^ in India [6]. However, urban elderlies reported illnesses at a higher rate (34.04%) than their rural counterparts (24.63%) [6]. This may be an implication of inaccessibility of healthcare and lack of social awareness which have been argued to blur individual’s conception of morbidity and its reporting thereof [7]. Studies have also found that in areas with inadequate access to healthcare, the morbidity level might actually reflect the level of healthcare utilization, rather than the actual health-status [8, 9]. Moreover, the data on treatment seeking behaviour among the elderly population indicated that a smaller share of ailing elderly in rural India (89%) sought medical treatment for an illness compared to their urban counterparts (96%) [6]. An overwhelming majority (80%) of the elderly with unmet needs of healthcare are concentrated in the rural areas [6]. With the setting up of newer health facilities at the grassroots level under the aegis of the National Rural Health Mission, 2005, an increase in the share of rural patients availing public health services has been observed [10]. This trend continued as rural elderly persons utilized public healthcare services at a higher rate (39.7%) in comparison to urban dwellers (25.5%) [6]. A higher share of elderly in rural areas (9%) cited ‘required specific services not available’ as a reason behind not availing government health facilities than urban residents (5%) [6]. Healthcare services in urban areas in India have been found to usually receive a larger share of public resources, resulting in lower investments in rural health infrastructure that suffers from issues of ill-management, absenteeism among health facility staff and lack of training for capacity building of health personnel [11, 12]. Majority of the ailing elderly, across rural and urban areas, did not avail government services and had to depend largely on the private health sector services mainly due to unsatisfactory quality of government services or long waiting involved even when quality was satisfactory [6]. Given this existing rural-urban disparity in the proportion of persons responded as ailing (PPRA) as well as utilisation of healthcare, it, therefore, becomes a matter of great importance to investigate whether all elderly persons with similar health needs are able to access the same set of health services of comparable quality irrespective of their geographies, demographic factors or socio-economic status [13].

Accessibility and utilisation are the two critical aspects of the efficiency of healthcare services, representing broadly the supply and demand sides of the healthcare delivery system respectively. While prior studies have established sub-national healthcare disparities due to inadequate bandwidth of existing infrastructure to serve the length and breadth of India [5, 10, 14–17], there has been a limited body of research work attempting the exploration of the determinants of the rural-urban gap in utilisation of healthcare. Moreover, many of the studies addressing the issue of the rural-urban inequalities are based on a small sample size, conducted locally at micro-level [18–24]. An important objective of India’s National Health Policy (2017) [25] is to *“progressively achieve universal health coverage”* which is posited upon mitigating the sub-national disparity that necessitates identifying the drivers of the disparity for targeted policy intervention. This study, therefore, aims to delve into the central question: what are the magnitude and contributory factors of rural-urban differential in health care utilisation among the elderly population in India who have reported some ailment in the past 15 days recall period? In this regard the study wants to test the socio-ecoconomic gradient hypothesis [5, 26, 27] that the health-seeking behaviour of the Indian elderly has positive associations with their Socio-Economic Status (SES).

In the following sections, we provide a description of the data source used for the study along with the variables and statistical techniques employed for the analysis. We then present the results of our analysis followed by a discussion on the findings and concluding remarks.

## Materials and methods

### Data source

Unit level data collected through the 75th round of the National Sample Survey (NSS) during July 2017 – June 2018, corresponding to the Schedule number 25.0 (Social Consumption: Health) [6] has been used for the present study. The NSS 75th round survey adopted a stratified multi-stage design. The survey collected nationally representative data on self-reported morbidity,^3^ utilisation of health care services and expenditure incurred on treatment for a specified recall period. Besides the detailed information pertaining to the respondent’s demographic and socio-economic characteristics, the survey also collected certain additional information with respect to the elderly population aged 60 years and above which have a bearing on their economic dependence, state of health and degree of isolation.

In the present study, out of the total sample size of elderly population (42,762 persons), only those elderly persons who self-reported some illness in the past 15 days recall period were considered for analyses related to healthcare utilization. This is because of the healthcare facilities are supposed to be utilised only by those who need it, i.e. suffer from any ailment that requires medical treatment. Thus, the number of observations for analyses pertaining to utilisation of healthcare was restricted to only 13, 674 (6615 in case of rural and 7059 in case of urban). It is to be noted that the figures do not pertain to number of ailing persons, rather number of spells of ailment reported. This means that there may be more than one spell of ailment reported by an individual which have been included as separate observations because treatment must be sought for each incidence of illness by every individual. The socioeconomic and demographic profile of the samples in the age-group 60 years and above pertaining to the 75th round is presented in Appendix 1.

### Variables for statistical analyses

#### Dependent variable

**Fig. 1:**
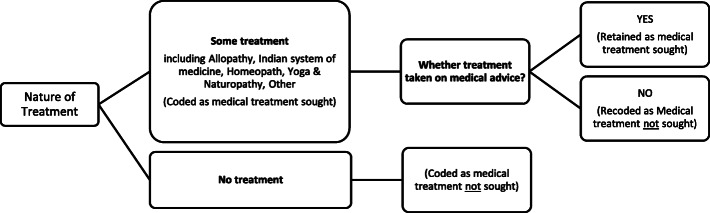
Question tree used to collect data on treatment-seeking behaviour (NSS, 2019)

*Whether any treatment taken on medical advice* (Yes = 1, No = 0) has been chosen as the dependent variable. In the 75th round of NSS, information was collected on the nature of treatment sought for the spells of ailment^4^ during the last 15 days recall period. This is inclusive of treatment received for ailments (chronic and short-term) that started within the past 15 days as well as those that started more than 15 days ago but continued during the recall period. In this study, all the categories of treatment except 'no treatment' were clubbed together, thereby getting binary category- treatment sought (1), no treatment sought (0). Further, cases of reported self-treatment were re-categorized in the 'no treatment' category. Figure 1 illustrates the sequence of questions asked to collect data on treatment received from individuals who reported suffering from an ailment any time during the 15 days preceding the date of survey.

#### Predictor variable

##### Place of residence

This variable was categorised as urban and rural. Rural India is comparatively less developed than urban India with poor public investment in healthcare infrastructure. Thus, accessibility of health care services especially geriatric care is an issue of concern in rural India [5, 28, 29].

#### Covariates

Four broad domains of covariates have been identified that may induce inequalities in health-care utilization across rural and urban parts of India. These domains pertain to sets of demographic factors, socio-economic factors, social support/ institutional factors and geographical factors.

The demographic variables include: (a) age: categorized as younger olds (60–69 years), old-olds (70–79 years) and oldest olds (80 years and above), due to the varying disease burden and health seeking behaviour of the age cohorts, age-squared as a continuous variable has been used in the regression model; (b) sex (male and female); (c) social groups^5^ (Scheduled Castes (SC), Scheduled Tribes (ST), Other Backward Classes (OBC) and others); (d) marital status (currently married and others (inclusive of those who never married or are divorced/ separated/ widowed)); and (e) religion (Hindu, Muslim, and others (comprising all other religious identities like Christianity, Sikhism etc.)).

The socio-economic variables comprise of (a) educational status: the NSS collected information on the general educational level of individuals in 15 categories that were classified into four broad categories vis a vis illiterate; upper primary or below; secondary; higher secondary and above for the present study (b) economic dependence (economically independent, partially dependent and fully dependent) (c) economic group: given that NSS provides data on households’ monthly consumption expenditure and not income, Monthly Per Capita Consumption Expenditure^6^ (MPCE) has been used as a proxy for income. MPCE quartiles were created based on the relative ranking of the households as per standard of living vis a vis poor, lower-middle, upper-middle and rich; (d) household size (categorized as 5 or less and more than 5, given the mean household size of the sample is 5), household size-squared as a continuous variable has been used in the regression model.

The social support variable comprised living arrangement (living alone and other arrangements like living with spouse and/or children and/or other relatives and/or non-relations clubbed together). The institutional variable comprised of health insurance (categorized as insured and not insured). Lastly, the geographical variable included region^7^ (North, East, West, Central, Northeast and South) to account for the effect of the regional imbalance in India.

### Statistical analyses

Bivariate percentage distribution (cross-tabulation) is calculated to estimate the differentials in the rate of seeking treatment (by ailing persons) on medical advice by predictor variables. The results are tested for statistical significance by using Pearson’s Chi-squared test for homogeneity or independence. The sample data were weighted to reflect the structure of Indian population using the formula provided in the report of National Sample Survey (2019) [6].

Two binary logistic models have been proposed to capture the crude and the adjusted association between health seeking behaviour and place of residence (rural/ urban). The model on adjusted association between healthcare utilisation and place of residence controls for a vector of demographic, socio-economic, social support, institutional and regional variables and examines the complex interplay of these variables in determining the magnitude and direction of rural-urban differentials in utilisation of healthcare by the elderly population in India. The results are presented as crude (cOR) and adjusted odds ratios (aOR) with 95% confidence intervals (CI). The binary logistic model used to examine the association between healthcare utilisation and predictor variables can be expressed by the following equation [31]:

1 $$ {\mathrm{P}}_i=\mathit{\Pr}\left(Y=1|X={x}_i\right)=\frac{\exp \left({\beta}_o+{x}_i{\beta}_n\right)}{1+\exp \left({\beta}_o+{x}_i{\beta}_n\right)}+u $$

*P*_*i*_ is the propability of utilising healthcare, *x*_*i*_ is the vector of socio-economic covariates of *i*^*th*^ individual, the coefficients *β*_*n*_ are parameters to be estimated an *u* is the error term. The odds ratios, OR are computed as (*P*_*i*_/1- *P*_*i*_).

Finally, to compute the group differences (rural and urban) in the rate of healthcare utilization among the elderly population in India and to decompose these differences into the major contributing factors, Fairlie’s decomposition method [32, 33] which is a non-linear approximation of the Blinder-Oaxaca decomposition technique (1973) [34, 35] has been employed. The average difference in the rates of heath care utilisation between rural and urban elderly persons may be expressed as (Fairlie, 1999):

$$ {\overline{Y}}^R-{\overline{Y}}^U=\left[{\sum \limits}_{i=1}^{N^R}\frac{F\left({X}_i^U{\overset{\frown }{\beta}}^R\right)}{N^R}-{\sum \limits}_{i=1}^{N^U}\frac{F\left({X}_i^U{\overset{\frown }{\beta}}^U\right)}{N^U}\right]+\left[{\sum \limits}_{i=1}^{N^U}\frac{F\left({X}_i^U{\overset{\frown }{\beta}}^R\right)}{N^U}-{\sum \limits}_{i=1}^{N^U}\frac{F\left({X}_i^U{\overset{\frown }{\beta}}^U\right)}{N^U}\right] $$

Where,

${\overline{Y}}^R and\ {\overline{Y}}^U$ *are* the average probability of the binary outcome, i.e. rate of healthcare utilisation for rural and urban elderly population respectively,

*N*^*R*^ and *N*^*U*^ are the sample sizes for rural and urban elderly population respectively,

*F* is the cumulative distribution function of logistic distribution,

${X}_i^R\  and\ {X}_i^U$ are the row vectors of average values of the independent variables, and.

${\hat{\beta}}^R\  and\ {\hat{\beta}}^U$ are the vectors of coefficient estimates for rural and urban elderly population respectively.

The first term in brackets, in the above equation, represents the part of the rural-urban gap in health care utilisation that is due to the differences in the characteristics of the two groups, constituting the relative contribution of each of the observed predictor variables. The second term represents the degree to which rural and urban elderly population with similar observable characteristics have different rates of healthcare utilisation and also captures the portion of the rural-urban gap due to group differences in unmeasurable or unobserved endowments, constituting the ‘unexplained’ or residual part of the differences. The decomposition has been undertaken using the pooled estimated coefficients of the two groups- rural and urban.

All the statistical analyses were conducted using the software STATA version 14.

## Results

### Rural-urban differential in health care utilisation among elderly population

The rural-urban differential of rate of seeking treatment among the elderly population (aged 60 years and above) in India by select background characteristics is presented in Table 1. At the all India level, 92% of the ailing elderly population reported to have sought treatment on medical advice for an episode of illness. However, a smaller share of ailing elderly in rural India (89%) sought medical treatment compared to their urban counterparts (96%). The distribution of rate of seeking medical treatment across different demographic variables indicated that elderly persons who are aged between 80 years and above have the highest rate of healthcare utilization compared to the younger-olds (60–69 years) and the old-olds (70–79 years). Likewise, higher percentage of male elderly persons have sought medical treatment in comparison to the female elderly persons across rural and urban India. While OBCs had the highest rate of healthcare utilization among all the caste groups at the overall country level and in rural India, ‘other’ castes had the highest treatment seeking rate in urban India. The rural-urban difference, disfavoring those residing in rural India is the widest among Scheduled Tribes with a rural/urban ratio of 0.87. Never married, widowed, divorced or separated older persons had a lower rate of treatment seeking than the currently married ones. Also, with respect to religion, Hindu elderly had the least rate of healthcare utilization compared to Muslims and other religious groups.

**Table 1:** Rural-Urban Differential in Health Care Utilisation among Elderly Population by Select Background Characteristics in India (2017–18)

		Total		Rural		Urban		
		Rate of Seeking Medical Treatment (%)	*P* value	Rate of Seeking Medical Treatment (%)	*P* value	Rate of Seeking Medical Treatment (%)	*P* value	Rural/Urban Ratio
Place of Residence	Rural	88.99	†		†		†	
	Urban	96.07						
Age	Younger old (60–69 years)	92.17	†	90.01	†	95.29	†	0.94
	Old-old (70–79 years)	90.81		86.56		97.03		0.89
	Oldest old (80 years and above)	93.42		90.30		97.56		0.93
Sex	Male	92.51	†	89.8	†	96.32	†	0.93
	Female	91.32		88.25		95.84		0.92
Social Group	ST	81.7	†	80.41	†	92.1	†	0.87
	SC	91.03		89.44		94.47		0.95
	OBC	92.51		90.64		95.79		0.95
	Others	92.5		88.07		96.68		0.91
Marital Status	Currently Married	92.89	†	90.06	†	96.68	†	0.93
	Others	90.38		87.49		95.03		0.92
Religion	Hindu	90.93	†	87.87	†	95.52	†	0.92
	Muslim	94.77		92.93		97.03		0.96
	Others	96.57		94.57		99.03		0.95
Education	Illiterate	88.02	†	85.95	†	94.63	†	0.91
	Upper Primary or Below	93.97		92.07		96.61		0.95
	Secondary	95.99		93.43		97.4		0.96
	Higher Secondary or above	96.32		97.97		95.93		1.02
Economic Dependence	Not Dependent	93.75	†	91.27	†	96.48	†	0.95
	Partially dependent	91.27		89.24		94.86		0.94
	Fully dependent	91.14		87.79		96.27		0.91
Economic Status	Poor	86.65	†	86.12	†	90.61	†	0.95
	Lower Middle	91.26		89.84		95.27		0.94
	Upper Middle	93.54		91.14		96.37		0.95
	Rich	96.19		92.97		96.98		0.96
Household Size	5 or less	92.29	†	89.05	†	96.58	†	0.92
	More than 5	91.06		88.88		94.81		0.94
Living arrangement	Alone	87.15	†	83.44	†	94.42	†	0.88
	With spouse and/or other members	92.26		89.48		96.18		0.93
Health Insurance	Not insured	91.1	†	87.81	†	95.62	†	0.92
	Insured	93.79		91.64		97.25		0.94
Region	Northeast	70.46	†	64.88	†	90.68	†	0.72
	South	95.08		93.47		97.64		0.96
	West	93.75		86.94		98.41		0.88
	North	91.55		89.47		94.08		0.95
	Central	88.43		87.88		89.76		0.98
	East	87.52		83.29		94.44		0.88
TOTAL		**91.9**		**88.99**		**96.07**		0.93

Note: † *p* < 0.001

Source: Author’s calculation from the NSS 75th Round on Social Consumption: Health

The distribution of rate of health care utilization across different socio-economic groups showed that the elderly persons with some level of education utilized health care at a higher rate than the illiterate older persons. There is a 9-percentage point difference between the rates of health care utilization of the rural illiterates and the urban illiterates, in favour of the urban. Also, the economically independent older persons and those belonging to the richest wealth quartile had a higher rate of seeking medical treatment than those who are fully or partially dependent economically or belong to the poorest wealth quartiles. Older persons living alone or in a larger household (sized more than 5) seek medical treatment for an illness at a lower rate than those living with spouse and/or other members and in a household with size of 5 or less, respectively. There is an 11-percentage point difference between the rates of health care utilization of the older persons living ‘alone’ in rural and urban areas, in favour of the urban. This distribution pattern is uniform across rural and urban India wherein older persons residing in rural India, regardless of their demographic, social or economic attributes, have a lower rate of treatment seeking when compared to their urban counterparts. However, there is one deviation from this general pattern in case of elderly with education of ‘higher secondary or above’ residing in rural India having a higher rate of treatment seeking than their urban counterparts with the same level of education. Older persons who have health insurance have a higher rate of treatment seeking than those who are not insured in both urban and rural India.

The rural-urban differential in rate of healthcare utilization is consistently in favour of the elderly persons residing in urban India across various regions of India. While the rural-urban difference is the widest in case of Northeast region (rural-urban ratio of 0.72), this difference is the smallest in Central and Southern India (rural/urban ratio of 0.98 and 0.96 respectively).

### Rural-urban differences in disease burden and untreated morbidities

The most common ailment reported by the urban and rural elderlies alike were hypertension and diabetes. However, hypertension was most common in rural area while diabetes was the most reported ailment in urban areas. Table 2 presents the varying disease burden of the elderly population in India by age and place of residence (rural/ urban). The top ten most reported ailments constitute roughly 82% and 84% of the total disease burden in rural and urban India respectively. Table 3 presents the rural-urban differential of the unmet need of healthcare. Of all the ailing elderly persons in India that received no treatment, an overwhelming majority (70%) belonged to the rural area. Further, the ailing elderly people who sought treatment but not on medical advice also majorly belong to the rural India (63.4). Likewise, the majority of the sick elderly persons who sought treatment from informal healthcare service providers^8^ are concentrated in the rural areas (69.6%). Table 4 gives the distribution of the untreated morbidities among the elderly population for rural and urban India separately. Joint and bone disease was the most neglected ailment in rural areas while diabetes was the most commony untreated ailment in urban areas. The top-ten listed untreated morbidities together constitute roughly 74% and 76% of the total untreated morbidities in rural and urban areas respectively.

**Table 2:** Distribution of reported morbidities by age and place of residence among the Elderly Population in India (2017–18)

RURAL				
Reported diagnosis/ symptoms	Younger Old (60–69 years)	Old-Old (70–79 years)	Oldest Old (80 years and above)	Entire older population (60 years and above)
Hypertension	23.24	23.11	24.19	23.31
Diabetes	20.45	19.3	17.95	19.82
Joint or bone disease	10.86	9.29	11.05	10.42
Heart disease^a^	7.89	8.74	8.45	8.21
Asthma	4.8	6.81	7.15	5.67
Feversb^b^	5.47	5.49	4.29	5.34
Back or body aches	2.81	3.1	2.21	2.83
Pain in abdomen^c^	2.99	2.23	3.25	2.8
Weakness in limb muscles and difficulty in movements	1.83	1.42	1.95	1.72
Neurological^d^	1.21	2.03	2.34	1.59
URBAN				
Reported diagnosis/ symptoms	Younger Old (60–69 years)	Old-Old (70–79 years)	Oldest Old (80 years and above)	Entire older population (60 years and above)
Diabetes	30.34	26.96	25.65	28.83
Hypertension	26.16	28.18	27.77	26.93
Heart disease^a^	6.84	8.56	9.71	7.66
Joint or bone disease	7.22	7.34	6.6	7.18
Asthma	3.37	4.31	4.48	3.77
Fevers^b^	3.28	2.79	1.62	2.95
Back or body aches	2.02	2.01	1.49	1.95
Pain in abdomen^c^	1.61	1.52	1.99	1.63
Goitre and other diseases of the thyroid	1.88	1.22	0.62	1.54
Others^e^	1.52	1.52	2.12	1.59

Note: ^a^ includes Chest pain, breathlessness

^b^ includes: Typhoid, fever with rash/ eruptive lesions and fevers of unknown origin, all specific fevers that do not have a confirmed diagnosis; excludes: fever with loss of consciousness or altered consciousness, Malaria, fever due to Diphtheria, Whooping Cough

^c^ includes Gastric and peptic ulcers/ acid reflux

^d^ includes Stroke/ hemiplegia/ sudden onset of weakness or loss of speech in half of body

^e^ Symptom not fitting into any of the categories given

Source: Author’s calculation from the NSS 75th Round on Social Consumption: Health

**Table 3:** Rural-urban differential of no treatment, non-medical treatment and treatment from informal healthcare service providers among the Elderly Population in India (2017–18)

	RURAL (%)	URBAN (%)
No treatment sought	70.00	30.00
Non-medical treatment sought^a^	63.44	36.56
Treatment sought from Informal healthcare service provider	69.61	30.39

Note: ^a^ includes self treatment, treatment on consultation from other household member or friend, treatment on consulting medicine shop, others

Source: Author’s calculation from the NSS 75th Round on Social Consumption: Health

**Table 4:** Distribution of untreated morbidities by place of residence among the Elderly Population in India (2017–18)

RURAL						
Reported diagnosis/ symptoms	No treatment	Non-medical treatment			Treatment from informal healthcare service provider	TOTAL
		Self/ household member/ friend	Medicine shop	Others		
Joint or bone disease	25.17	28.38	18.07	13.56	15.49	21.66
Fevers ^a^	4.08	8.78	17.47	6.78	12.68	10.32
Hypertension	6.12	14.19	6.02	16.95	14.08	10.15
Back or body aches	4.76	6.08	10.24	1.69	4.23	6.26
Asthma	6.12	3.38	5.42	8.47	5.63	5.41
Diabetes	6.8	4.73	1.81	8.47	8.45	5.25
Acute upper respiratory infections ^b^	4.76	4.73	6.02	3.39	2.82	4.74
Pain in abdomen ^c^	2.72	4.73	4.22	3.39	8.45	4.4
Weakness in limb muscles and difficulty in movements	3.4	4.05	1.81	3.39	2.82	3.05
Fever due to Diptheria, Whooping Cough	0.68	3.38	3.61	1.69	4.23	2.71
URBAN						
Reported diagnosis/ symptoms	No treatment	Non-medical treatment			Treatment from informal healthcare service provider	TOTAL
		Self/ household member/ friend	Medicine shop	Others		
Diabetes	12.7	22.06	12.84	28.95	6.45	16.18
Joint or bone disease	15.87	13.24	11.93	13.16	22.58	14.24
Hypertension	15.87	11.76	8.26	23.68	16.13	13.27
Fevers ^d^	1.59	14.71	15.6	2.63	6.45	10.03
Back or body aches	12.7	1.47	5.5	0	6.45	5.5
Acute upper respiratory infections ^b^	1.59	5.88	9.17	0	3.23	5.18
Fever due to Diptheria, Whooping Cough	1.59	4.41	3.67	2.63	0	2.91
Headache	0	5.88	3.67	0	3.23	2.91
Weakness in limb muscles and difficulty in movements	3.17	1.47	0.92	7.89	6.45	2.91
Asthma	1.59	0	6.42	0	3.23	2.91

Note: ^a^ includes Typhoid, fever with rash/ eruptive lesions and fevers of unknown origin, all specific fevers that do not have a confirmed diagnosis; excludes Malaria, fever with loss of consciousness or altered consciousness

^b^ includes cold, runny nose, sore throat with cough, allergic colds

^c^ includes Gastric and peptic ulcers/ acid reflux/ acute abdomen

^d^ includes Typhoid, fever with rash/ eruptive lesions and fevers of unknown origin, all specific fevers that do not have a confirmed diagnosis; excludes Malaria

Source: Author’s calculation from the NSS 75th Round on Social Consumption: Health

### Rural-urban differentials in the use of health facilities

Public facilities were the most commonly utilised healthcare services accounting for roughly 40% of the services utilised by the elderly in rural India. In contrast, urban elderly people mostly avail the services of the private doctors/ clinics (43.5%). However, utilisation of the private sector facilities (comprising both private hospitals and private doctors/ clinics) outweighed use of the rest of the sources of healthcare services in both rural and urban areas accounting for 58% and 72% respectively. Nevertheless, the urban older population relies on the private sector facilities more than their rural counterparts (Table 5). Preference for a trusted doctor/ hospital and unsatisfactory quality of services in public facilities were the two most commonly cited reasons for not availing healthcare services from government sources in both urban and rural areas albeit constituting a varying proportion (Table 6).

**Table 5:** Use of different health facilities by place of residence among the Elderly Population in India (2017–18)

Type of Health Facility	RURAL (%)	URBAN (%)
Government/ Public Hospital (incl. HSC/PHC/CHC etc.)	39.69	25.49
Charitable/Trust/NGO run hospital	0.9	1.63
Private Hospital	25.9	28.91
Private doctor/ clinic	31.87	43.45
Informal health care provider	1.64	0.52

Source: Author’s calculation from the NSS 75th Round on Social Consumption: Health

**Table 6:** Reason for not availing government health facility by place of residence among the Elderly Population in India (2017–18)

Reason for not availing government health services	RURAL (%)	URBAN (%)
Required specific services not available	8.81	5.1
Available but quality not satisfactory	27.79	22.77
Quality satisfactory but facility too far	10.52	5.92
Quality satisfactory but involves long waiting	17.47	21.55
Financial constraint	0.46	0.2
Preference for a trusted doctor/hospital	29.17	40.73
Others	5.78	3.74

Source: Author’s calculation from the NSS 75th Round on Social Consumption: Health

Table 7 presents the rural-urban differential in accessibility of health facilities among the elderly population in India. Roughly 44% of the ailing older population in the rural India had to seek treatment from a facility in the urban area in the same district of residence. Further, 4.4% of the rural ailing elderly persons also had to travel to an urban facility located in a different district to seek treatment for their illnesses. This is in contrast to the 91.6% ailing elderly in the urban areas who received treatment for their illnesses at the place of their residence.

**Table 7:** Rural-urban differential in Place of treatment among the Elderly Population in India (2017–18)

Place of treatment	Same district (rural area)	Same district (urban area)	Within state different district (rural area)	Within state different district (urban area)	Other state
RURAL	49.93	43.53	0.91	4.41	1.22
URBAN	4.05	91.55	0.33	3.04	1.02
Total	26.23	68.34	0.61	3.7	1.12

Source: Author’s calculation from the NSS 75th Round on Social Consumption: Health

### Determinants of inequalities in health care utilisation among elderly population

In this section, the crude and adjusted odds ratios have been computed through logistic regression to assess the effect of place of residence on the health seeking behaviour of the ailing elderly population in India. The results of the logistic regression have been presented in Table 8. The crude analysis in model 1 indicated that the odds of seeking medical treatment for urban elderly persons is 3 times higher than their rural counterparts and this difference is highly significant (at 99% confidence level).

**Table 8:** Determinants of inequalities in health care utilisation among elderly population in India (2017–18)

Model Specifications	Model 1	Model 2
Number of observations	13,674	13,673
Wald chi2(1)	48.09	148.07
Prob > chi2	0.000	0.000
Pseudo R2	0.0315	0.0843
Log pseudolikelihood	-1.37E+ 09	-1.29E+ 09
Dependent Variable Medical Treatment Sought- Yes:1, No:0		
Covariates		**Odds Ratio** **(95% CI)**
Model 1		
Place of Residence	Rural®	**cOR**
	Urban	3.025† (2.212–4.136)
Model 2		
Place of Residence	Rural®	**aOR**
	Urban	2.139† (1.467–3.119)
Age Squared		1.000 (0.998–1.004)
Sex	Female®	
	Male	0.807 (0.542–1.202
Social Group	ST®	
	SC	1.713 (0.853–3.443)
	OBC	1.435 (0.769–2.678)
	Others	1.164 (0.584–2.318)
Marital Status	Others®	
	Currently Married	1.128 (0.756–1.683)
Religion	Muslim®	
	Hindu	0.495** (0.283–0.864)
	Others	1.078 (0.508–2.288)
Education	Illiterate®	
	Upper Primary or Below	1.704*** (1.148–2.529)
	Secondary	1.962** (1.069–3.602)
	Higher Secondary or above	2.043** (1.124–3.712)
Economic Dependence	Fully dependent®	
	Partially dependent	1.083 (0.710–1.652)
	Not Dependent	1.287 (0.837–1.980)
Economic Status	Poor®	
	Lower Middle	1.169 (0.744–1.837)
	Upper Middle	1.198 (0.708–2.028)
	Rich	1.307 (0.716–2.383)
Household Size Squared		1.001 (0.996–1.007)
Living arrangement	Alone®	
	With spouse and/or other members	1.798* (0.953–3.392)
Health Insurance	Not insured®	
	Insured	1.161 (0.801–1.681)
Region	Northeast®	
	South	6.653† (2.630–16.831)
	West	4.886*** (1.736–13.756)
	North	3.360** (1.275–8.858)
	Central	3.554*** (1.389–9.098)
	East	2.786** (1.105–7.022)

Note:**®**Reference category, † *p* < 0.001, *** *p* < 0.01 ** *p* < 0.05 and * *p* < 0.1

Source: Author’s calculation from the NSS 75th Round on Social Consumption: Health

In model 2, controlling of the effect of a range of demographic, socio-economic, social support, institutional and regional covariates, did not change the pattern of rural-urban differential in rate of treatment seeking. However, the inclusion of the covariates in the model, resulted in a contraction of the magnitude of the rural-urban differences. The odds of urban elderly seeking medical treatment remained very high nonetheless, i.e. twice that of their rural counterparts, significant at 99% confidence level.

Further, religion, education level and living arrangement emerged as statistically significant determinants of health care utilization among the elderly population India. Hindu elderly had almost 50% lower odds of utilizing health care than the Muslims (significant at 95% confidence level). An elderly with educational attainment of higher secondary or above was twice as likely as an illiterate elderly to seek medical treatment for an episode of illness (significant at 95% confidence level). Also, an elderly living with their spouse and/or any other member were 70% more likely to seek medical treatment than those living alone (significant at 90% confidence level). The regional variable was also a significant determinant of an elderly person’s rate of treatment seeking. With respect to the elderly residing in the Northeast region, elderly persons living in all other regions of India- Southern, Western, Northern, Central and Eastern regions, had higher odds of seeking treatment for an illness. The Southern region had the highest odds (6.65, significant at 95% confidence level) while the Eastern region had the lowest odd (2.79, significant at 95% confidence level) compared to the Northeast region.

### Major contributory factors of rural-urban difference in health care utilization among elderly population: decomposition analysis

This section investigates the separate contributions from the rural-urban differences in each set of predictor variables to the rural-urban gap in the rate of seeking medical treatment for an illness among the elderly population in India by employing Fairlie’s decomposition technique (1999). The results of the decomposition analysis have been presented in Table 9. The rural and urban gap in healthcare utilization among the elderly population is − 0.0708, i.e. 7.1 percentage points higher for the urban elderly persons. The range of covariates considered in the model together explain 47.4% of the overall rural-urban gap, using the pooled estimated coefficients of the two groups- rural and urban. Using the rural coefficients as the reference, 39.3% of the gap could be explained while taking the urban coefficients as the reference, 35.1% of the differences could be explained (Appendix 2).

**Table 9:** Decomposition of the Rural-Urban Differential in Utilisation of Health Care among Elderly Population in India (2017–18)

	Healthcare Utilisation		
	Coefficient	%	***P*** > |z|
Rural Mean	0.8899		
Urban Mean	0.9607		
Rural-urban gap	−0.0708		
Contributions from rural-urban differences in:			
Age	0.00000043	−0.0006	0.999
Sex	0.0013	−1.80	0.371
Social Group	−0.0025	3.56	0.177
Marital Status	−0.0003	0.47	0.733
Religion	0.0001	−0.08	0.819
Education	−0.0160***	22.58	0.000
Economic Dependence	−0.0002	0.23	0.590
Economic Status	−0.0128**	18.10	0.016
Household size	−0.0000082	0.01	0.972
Living Arrangement	−0.0014	2.03	0.207
Health Insurance	−0.0001	0.10	0.697
Region	−0.0016**	2.20	0.018
All included Variables	**−0.0336**	**47.41**	
Unexplained gap	**−0.0372**	**52.59**	

Note: ** *p* ≤ 0.05, *** *p* ≤ 0.01

Source: Author’s calculation from the NSS 75th Round on Social Consumption: Health

The decomposition results reveal that the rural-urban differences in the socio-economic factors vis a vis education (*p* < 0.01) and economic status (*p* < 0.05) together contribute to roughly 41% of the overall rural-urban gap in utilisation of health care by the elderly population in India. Regional differences (*p* < 0.05) explain 2.2% of the rural-urban inequality. The rest of the covariates have statistically insignificant contribution to the rural-urban differential in healthcare utilisation. As per NSS (2019), 38% of the rural elderlies belong to the lowest wealth quartile (poor) while only 7% of the urban dwellers belong to the poorest wealth quartile. On the other hand, 49% of the urban elderly belong to the highest wealth quartile (rich) compared to only 8% of the rural elderly in the same category. This rural-urban disparity in the distribution of income translates into a large negative effect (− 0.0128) on the rate of healthcare utilisation of the rural elderly people. The rural-urban gap in economic status, thus, contributes to 18% of the rural-urban difference in health care utilisation. Moreover, low levels of education among the rural elderly in comparison to their urban counterparts shows a negative effect (− 0.0160) on their likelihood to seek medical treatment for an illness. In rural India, 55% of the older population is ‘not literate’^9^ as opposed to a much smaller proportion (25%) in the urban areas; while 24% of the urban dwellers have education level of higher secondary or above compared to only 4% of the rural elderly with the same level of education. This disparity in the level of educational attainment has a contribution of roughly 23% in explaining the rural-urban differential in health seeking behaviour among the older population in India. Thus, an improvement in the economic status and level of education among the rural elderly population has the potential to diminish the rural-urban gap in healthcare utilisation significantly.

The predictor variables- social group, living arrangement and sex also contribute 3.6%, 2% and 1.8% to the rural-urban gap, respectively. However, these contributions in explaining the rural-urban inequalities are not statistically significant. Marital status, religion, economic dependence, household size and health insurance have marginal contribution in the inequality between rural and urban dwellers but the results are statistically insignificant. Roughly 52.6% of the rural-urban difference remains unexplained due to ommitted variables.

## Discussion and conclusion

This paper made an attempt to fill a critical research gap in the domain of aging and health in India by quantifying and decomposing the rural-urban differential in the health care utilisation among older population aged 60 years and above in India. A number of intriguing findings were advanced by this study. The estimates of bi-variate analysis of socio-economic covariates showed that the urban dwellers have a higher rate of utilisation of health-care services when suffering from any ailment compared to the rural elderly. Results yielded by the logistic regression indicated that place of residence has a strong association with seeking treatment for an ailment among the elderly in India. Both the crude and adjusted odds ratios indicated that urban elderly have a much higher likelihood of seeking medical treatment than those living in rural India. This urban advantage has been established by the findings of a number of studies previously published [5, 14, 36–38]. In addition to place of residence, the covariates- education, religion, living arrangement and region also displayed strong associations with healthcare utilisation. Educational attainment, an important proxy of an individual’s socio-economic status (SES), has been an overarching determinant of a person’s health seeking behaviour as it reflects the ‘opportunities and privileges afforded to people within the society’ [36, 37, 39]. With increasing level of education, the likelihood of an older person utilising healthcare services increases significantly. Religion was also found to be a significant determinant of health seeking behaviour wherein Hindu elderly people had a lower likelihood of seeking treatment for an illness in comparison to Muslim elderly persons [36]. Despite the socio-economic status of Muslims in India being worse off than the Hindus on an average, Muslim advantage in health indicators, especially that of child survival has been well documented and labelled as a ‘puzzle’ or a ‘paradox’, however, very few studies have attempted to address this surprising trend [40]. The findings of a study on the health seeking behaviour of the adult population (aged 20–59 years) in India has found lower odds of Hindu adult population utilising healthcare for an episode of illness compared to Muslim adults, consistently over two periods 2014 and 2017–18 [41]. A probable explanation behind better health-seeking behaviour among Muslim elderly persons might be that the Muslim population is concentrated in districts with better public health facilities [42]. As per NSS (2019), 42% of India’s Muslim elderly population resides in the southern region with one of the highest likelihoods of healthcare utilisation among all the other regions in India, as shown in the regression results. Moreover, ‘Hindus’ in the present analysis refers to a homogenous group. As per NSS (2019), majority of the Scheduled Tribes (55%) are Hindus, located in the rural areas (64%) and have the lowest likelihood of seeking healthcare among all the social groups albeit the results being statistically insignificant. Poor quality of rural health infrastructure coupled by inaccesibility have been found to be the major barriers to utilising healthcare services by the tribal population [43]. However, the role of religion needs to be explored further to analyse the factors explaining the Muslim advantage in healthcare utilisation. Living arrangement is yet another important determinant of an older person’s healthcare utilisation. Our study resonates with previous studies that have found that older persons living with their spouse or children or any other relative/ non-relative are more likely to seek medical treatment than those living alone in traditional societies like India where changing inter-generational relations is impacting the well-being of the elderly [44–47].

While the results of the regression clearly established the strong association between place of residence and likelihood of healthcare utilisation, the exercise of Fairlie's decomposition shed light on the major contributory factors that determine this rural-urban differential. The decomposition results suggested considerable rural-urban inequality disfavouring the rural dwellers and health care utilisation was found to be 7% higher among the older population residing in urban India than their rural counterparts. Level of education and economic status, both of which are indicators of a person’s SES, were the two major determinants of the existing rural-urban differential in healthcare utilisation, together constituting 41% of the existing rural-urban differential. Poor economic status was observed to be a source of inducing and expanding inequality in healthcare utilisation while better educational attainment could contract the magnitude of inequalities. Compelled by illiteracy and poverty, elderly in the rural areas constitute a very large share (84%) of the total elderly workforce [2, 48]. The rural elderly workers are mainly self-employed in agricultural sector and allied activities without any retirement age or pension benefits [2, 48]. Low-level of earnings during younger ages that results in scanty savings among the elderly has been flagged as an important push factor behind elderly labour-force participation [49]. However, even when engaged in paid work, roughly 55% of the elderly workforce is still economically dependent on others, either fully or partially in both rural and urban areas. The poor SES of the elderly in general and rural elderly in particular, when juxtaposed with increasing need for medical care with progressing age, renders them as extremely vulnerable in a country like India with a paralysed system of social safety net. Furthermore, regional factor has a minor but significant (statistically) contribution in explaining the rural-urban gap, given the widespread regional imbalance and heterogeneity in India [50].

The findings of this study are in tune with previous studies on health inequities [5, 26, 27, 50–53] pertaining to both developed and developing countries that have affirmed the applicability of the social gradient hypothesis with findings suggesting that the level of health care utilisation is distributed along the social gradients. A number of studies have provided evidence that a person with lower level of educational attainment and income is less likely to have access to health care services and vice versa [54–56]. The present study confirms the same. Financial empowerment has the potential to diminish the existing inter-group disparities among the elderly population in developing societies like India [57].

The report of the CSDH (2008) observes that concerted efforts in the realms of social, economic and political would lead to dramatic narrowing of the health differences that exist between and within countries [58]. In conclusion, it can, therefore, be said that by reducing the existing socio-economic and regional inequalities, the rural-urban inequalities in healthcare utilisation can be curbed. In order to improve the health care utilisation by the population across all socio-economic and demographic groups, enhancement in accessibility to health care services in addition to expansion in health care infrastructure, particularly the public facilities is needed. The rural and poorer elderly have a greater share of untreated morbidities due to lack of access and means to utilise health care services. Public health care services even when available are perceived to have poor quality of services [6]. Due to the poor quality of services, ailing persons have to resort to utilise private healthcare services putting a huge burden on their expenses [59–61]. Moreover due to lack of awareness, majority of the rural elderly do not avail any of the geriatric welfare services available near their homes [62, 63]. Thus, public health care provisions need to strengthen both in terms of quality and outreach by way of greater public investments in the health sector. The availability of geriatric out-patient departments is limited to tertiary care facilities that are concentrated mostly in urban areas [28] which needs to be addressed by building advanced health infrastructure in the rural areas. A strong social security system (old age/ widow pensions, government health insurance, etc.) needs to come into place to reduce the vulnerability of the poorer population. Implementation of poverty alleviation programmes and ensuring social-security of the elderly are also indispensable in bringing about equity in healthcare utilisation. Any health policy must recognise that an individual’s health outcomes are intricately linked to other aspects of their life and to their position in the socio-economic gradient. An integrated health policy employing the life-cycle approach is the need of the hour in achieving a holistic and equitable health seeking behaviour.

Limitation of the study: The study is based on self-reported data on healthcare utilisation which is prone to subjectivity of perception and reporting bias. The rate of seeking medical treatment is more than 80% across all demographic, socio-economic and geographical sub-categories. This figure somewhat raises suspicion over the reliability of data. Self-reporting of whether or not medical treatment was sought may have been over-reported. The absence of any mechanism for cross-checking the reported information for majority of the ailments, barring a few through medical prescriptions/ entry in public clinic registers, diagnostic records, etc. posed as a limitation of the present study.

The NSS has a list of over 60 categories of reported diagnosis and/or main symptom. Many of these categories are broad, eg. “pain in abdomen (including gastric and peptic ulcers/ acid reflux)”; “cancers (known as well as suspected by physician) and any growing painless lump in the body”, etc. There is an absence of any severity measure of the diseases. For example, a gowing lump may or may not be malignant in nature. Also, a cataract may be immature, mature or hyper mature. Since cataract surgery is an elective surgery, an immature cataract if postponed for a few weeks may not be as harmful as a neglected hyper mature blinding cataract. There is no data describing the distinctions in severity. Also, the adequacy of the treatment received is difficult to quantify in the absence of any related data. In view of these data gaps, the quantification of the degree of danger that a lack of treatment poses, is difficult to achieve in a robust manner.

## Data Availability

Yes - all data are fully available without restriction. The present study is based on secondary data (National Sample Survey, India) which is available in the public domain. The data can be accessed from: http://mospi.nic.in/unit-level-data-report-nss-75th-round-july-2017-june-2018-schedule-250social-consumption-health

## Appendix 1

**Table 10:** Percentage distribution of ailing elderly population sample by select background characteristics (Rural-Urban wise)

		Total		Rural		Urban	
		Frequency^a^	%	Frequency^a^	%	Frequency^a^	%
Place of Residence	Rural	6615	48.38				
	Urban	7059	51.62				
Age	Younger old (60-69 years)	8089	59.16	3877	58.61	4212	59.67
	Old-old (70-79 years)	4013	29.35	1969	29.77	2044	28.96
	Oldest old (80 +)	1573	11.50	769	11.63	803	11.38
Sex	Male	6844	50.05	3293	49.78	3551	50.3
	Female	6829	49.94	3321	50.2	3508	49.7
Social Group	ST	633	4.63	403	6.09	230	3.26
	SC	1666	12.18	1045	15.8	621	8.8
	OBC	5199	38.02	2733	41.32	2466	34.93
	Others	6176	45.17	2434	36.8	3742	53.01
Marital Status	Currently Married	8976	65.64	4290	64.85	4686	66.38
	Others	4698	34.36	2325	35.15	2373	33.62
Religion	Hindu	9970	72.91	4777	72.21	5193	73.57
	Muslim	1992	14.57	888	13.42	1104	15.64
	Others	1712	12.42	950	14.36	762	10.79
Education	Illiterate	5170	37.81	3353	50.69	1817	25.74
	Upper Primary or Below	4851	35.48	2405	36.36	2446	34.65
	Secondary	1569	11.47	515	7.79	1054	14.93
	Higher Secondary or above	2084	15.24	342	5.17	1742	24.68
Economic Dependence	Not Dependent	3650	26.69	1528	23.1	2122	30.06
	Partially dependent	3059	22.37	1697	25.65	1362	19.29
	Fully dependent	6965	50.94	3390	51.25	3575	50.64
Economic Status	Poor	2557	18.7	2013	30.43	544	7.71
	Lower Middle	3375	24.68	2156	32.59	1219	17.27
	Upper Middle	3659	26.76	1684	25.46	1975	27.98
	Rich	4083	29.86	762	11.52	3321	47.05
Household Size	5 or less	8128	59.44	3794	57.35	4334	61.4
	More than 5	5546	40.56	2821	42.65	2725	38.6
Living arrangement	Alone	422	3.09	223	3.37	199	2.82
	With spouse and/or other members	13,252	96.91	6392	96.63	6860	97.18
Health Insurance	Not insured	9407	68.79	4530	68.48	4877	69.09
	Insured	4267	31.21	2085	31.52	2182	30.91
Region	Northeast	328	2.4	182	2.75	146	2.07
	South	5579	40.8	3006	45.44	2573	36.45
	West	1922	14.06	601	9.09	1321	18.71
	North	2098	15.34	1075	16.25	1023	14.49
	Central	1393	10.19	670	10.13	723	10.24
	East	2354	17.22	1081	16.34	1273	18.03
TOTAL		13,674	100	6615	48.38	7059	51.62

Source: Author’s calculation from the NSS 75th Round on Social Consumption: Health

^a^unweighted sample size

## Appendix 2

**Table 11:** Decomposition of the Rural-Urban Differential in Utilisation of Health Care among Elderly Population in India (2017-18) using (a) rural coefficients and (b) urban coefficients as reference

**(a) Rural coefficients (reference)**	**Coefficient**	**%**	**P > |z|**
Total Difference (Rural - Urban)	−0.071		
Explained Difference	−0.028	39.30%	
Unexplained Difference	−0.043	60.70%	
**Contribution from rural-urban differences in:**			
Age	0.0008	−1.07	0.41
Sex	0.0012	−1.65	0.49
Social Group	−0.0025	3.47	0.234
Marital Status	0.0002	−0.27	0.916
Religion	0.0002	−0.24	0.608
Education	−0.0176***	24.85	0.004
Economic Dependence	−0.0003	0.46	0.542
Economic Status	−0.0060	8.52	0.322
Household Size	0.0001	−0.16	0.778
Living Arrangement	−0.0020	2.88	0.211
Health Insurance	−0.0002	0.21	0.656
Region	−0.0016**	2.21	0.021
**(b) Urban coefficients (reference)**			
Total Difference (Rural - Urban)	−0.071		
Explained Difference	−0.02	35.07%	
Unexplained Difference	−0.05	65.03%	
**Contribution from rural-urban differences in:**			
Age	0.0051**	−7.16	0.033
Sex	0.0003	−0.47	0.786
Social Group	−0.0025	3.59	0.563
Marital Status	−0.0007	1.05	0.688
Religion	−0.0005	0.68	0.183
Education	−0.0049	6.93	0.37
Economic Dependence	0.0003	−0.37	0.618
Economic Status	−0.0189*	26.76	0.053
Household Size	−0.0016	2.21	0.284
Living Arrangement	−0.0002	0.32	0.749
Health Insurance	−0.0003	0.42	0.311
Region	−0.0007	1.04	0.23

Source: Author’s calculation from the NSS 75th Round on Social Consumption: Health

* *p* ≤ 0.10, ** *p* ≤ 0.05, *** *p* ≤ 0.01

## Abbreviations

NSS: National sample survey
SC: Scheduled castes
ST: Scheduled tribes
OBC: Other backward classes
MPCE: Monthly per capita consumption expenditure
PPRA: Proportion of persons responded as ailing
SES: Socio-economic status
cOR: Crude odds ratio
aOR: Adjusted odds ratio

## Footnotes

1 As per NSS (2019), an ailment was identified as a chronic ailment if (a) the symptoms of the ailment persisted for more than one month on the date of survey, or (d) the course of treatment of the ailment, on medical advice, continued for a month or more on the date of survey.

2 Short-term ailments refer to all the non-chronic ailments, persisting for less than a month

3 In the NSS 75th round, the morbidity realated data was collected based on reporting of perceived ailments by the respondents, sometimes also identified with the help of the interviewer to whom they reported their symptoms. In case of a small set of ailments, a medical diagnosis was made a necessary condition for classifying a reported condition as diphtheria, whooping cough, TB, HIV/AIDS, cancers, diabetes, glaucoma and hypertension (NSS, 2019). The questions asked to collect this data on ailments included: (a) whether suffered from any communicable disease during last 365 days; (b) whether suffering from any chronic ailment; (c) whether suffered/ suffering from any other ailment (besides chronic ailment): i. any time during last 15 days, ii. on the day before the date of survey.

4 As per the NSS, an “ailment included (a) All types of injuries, such as cuts, wounds, haemorrhage, fractures and burns caused by an accident, including bites to any part of the body (b) Cases of abortion – natural or accidental. Ailments excluded (i) Cases of sterilisation, insertion of IUD, medical termination of pregnancy, etc. (ii) A state of normal pregnancy without complications (iii) Minor skin ailments, minor headaches and bodyaches, and minor gastric discomfort after meals (iv) Cases of pre-existing disabilities, unless a course of treatment of the disability on medical advice for a period of one month or more was continuing as on the date of survey.

5 The social groups are based on the persisting caste system in India, a form of hierarchical social stratification which constitutes an important axis of inequality in the Indian society [30].

6 The 75th round of the National Sample Survey collected data on “household’s usual monthly consumer expenditure (Rs.)”. To compute the per capita consumption expenditure, the household expenditure was divided by the household size. Thus, MPCE is the ratio of the total household consumer expenditure during a period of 30 days to the size of the household.

7 As per the administrative divisions of India, the regions have been defined as follows: North (comprising Chandigarh, Delhi, Haryana, Himachal Pradesh, Jammu and Kashmir, Ladakh, Punjab, Rajasthan, Uttarakhand and Uttar Pradesh); East (comprising Bihar, Jharkhand, Odisha, and West Bengal); West (comprising Dadra and Nagar Haveli and Daman and Diu, Goa, Gujarat, and Maharashtra); Central (comprising Madhya Pradesh and Chhattisgarh); Northeast (comprising Assam, Arunachal Pradesh, Manipur, Meghalaya, Mizoram, Nagaland, Tripura and Sikkim); and South (comprising Andhra Pradesh, Karnataka, Kerala, Puducherry, Tamil Nadu, Telangana and Andaman and Nicobar Islands, Lakshadweep).

8 This term covers a variety of health service providers who are untrained and work outside regulatory frameworks

9 As per NSS 75th round, a literate person is defined as a Person who can read and write with comprehension a simple message in any language.
